# Shift Work and Lifestyle Factors: A 6-Year Follow-Up Study Among Nurses

**DOI:** 10.3389/fpubh.2019.00281

**Published:** 2019-10-16

**Authors:** Hogne Vikanes Buchvold, Ståle Pallesen, Siri Waage, Bente E. Moen, Bjørn Bjorvatn

**Affiliations:** ^1^Department of Global Public Health and Primary Care, University of Bergen, Bergen, Norway; ^2^Norwegian Competence Center for Sleep Disorders, Haukeland University Hospital, Bergen, Norway; ^3^Department of Psychosocial Science, University of Bergen, Bergen, Norway

**Keywords:** shift work, night work, quick returns, health habits, lifestyle habits

## Abstract

**Objectives:** To evaluate different work schedules, short rest time between shifts (quick returns), and night shift exposure for their possible adverse effects on different lifestyle factors in a 6-year follow-up study.

**Methods:** Data stemmed from “The Survey of Shiftwork, Sleep and Health,” a cohort study of Norwegian nurses started in 2008/9. The data analyzed in this sub-cohort of SUSSH were from 2008/9 to 2015 and consisted of 1,371 nurses. The lifestyle factors were: Exercise (≥1 h/week, <1 h/week), caffeine consumption (units/day), smoking (prevalence and cigarettes/day), and alcohol consumption (AUDIT-C score). We divided the nurses into four groups: (1) day workers, (2) night workers, (3) nurses who changed toward, and (4) nurses who changed away from a schedule containing night shifts. Furthermore, average number of yearly night shifts (NN), and average number of quick returns (QR) were calculated. Paired *t*-tests, McNemar tests, and logistic regression analyses were used in the analyses.

**Results:** We found a significant increase in caffeine consumption across all work schedule groups and a decline in smoking prevalence for day workers and night workers at follow-up. Analyses did not show any significant differences between groups when analyzing (1) different work schedules, (2) different exposures to QR, (3) different exposures to NN on the respective lifestyle factor trajectories.

**Conclusion:** We found no significant differences between the different work schedule groups or concerning different exposures to QR or NN when evaluating these lifestyle factor trajectories. This challenges the notion that shift work has an adverse impact on lifestyle factors.

## Introduction

According to the last European Working Conditions Survey, 21% of the workforce is engaged in some type of shift work (1). Increased attention and research have been directed toward the possible adverse health effects of shift work during the last decades. In general, it has been shown that shift workers have elevated risks for a multitude of chronic diseases (2–4). Shift work has for instance been shown to be associated with increased risk of cardiovascular diseases (CVD), metabolic disturbances, and possibly some cancers (3, 5–9).

Models of the observed associations between shift work and chronic disease have primarily focused on two key pathways; behavioral and physiological changes and their reciprocal relationship (2, 4, 10). Shift work contributes to circadian disruption affecting hormonal systems regulating metabolism and stress responses, like glucose, and cortisol regulation (11–13). Night work disrupts the normal sleep-wake cycle giving rise to circadian misalignment interfering with both sleep duration and quality. Disturbed sleep is known as a risk factor for many diseases. For example, the CVD risk profile of shift workers mimics the risk profile for those with short sleep duration (4). Concerning psychosocial stress and social jet lag, shift work could potentially affect work-life balance with increased social and familial constraints. This could lead to difficulties initiating or maintaining lifestyle factors with positive health benefits. It has been hypothesized, although the results are inconsistent, that shift workers with schedules that include night work differ from day workers regarding lifestyle factors with adverse health consequences, for example in relation to smoking, alcohol, and exercise (14–18).

Night work duration and intensity are aspects of shift work believed to contribute to circadian stress and impaired work-life balance, possibly affecting lifestyle factors negatively. Short rest time between shifts (≤11 h), described as quick returns (QR), could also potentially influence lifestyle factors adversely through its association with stress, fatigue, and insomnia (19, 20). In addition, different work schedules might differ with respect to work-life balance impairment and thus possibly affect lifestyle factors.

The vast majority of previous studies on shift work in general and studies addressing shift work and lifestyle factors in particular have resorted to cross-sectional designs and limitations in the assessment of shift work exposure (e.g., shift work duration, night shift intensity, and type of shift work). The methodological limitations of shift work research have been addressed in several papers (5, 21–25). The need for large prospective studies with clearly defined shift work exposure parameters and, optimally, information about different aspects of shift work that might contribute to the increased risk of chronic disease have been emphasized.

This large prospective study of Norwegian nurses aimed to investigate different aspects of shift work and their impact on lifestyle factors. It was differentiated between different shift schedules (day work, night work, and changing of schedule toward- or away from night work). Different work schedules were evaluated for changes within—and between groups. Average yearly quick return exposure and average yearly night work exposure were also evaluated for a dose-response impact on the respective lifestyle factors. Specifically, we evaluated changes in exercise habits, caffeine consumption, smoking habits, and alcohol consumption. We hypothesized that work schedules containing night work, a high exposure to QR or a high exposure to night work would affect the examined lifestyle factors more adversely than schedules without these characteristics during the 6-year follow-up.

## Materials and Methods

### Design

The data in the present study stemmed from “The SUrvey of Shiftwork, Sleep and Health” (SUSSH), initiated in December 2008 (26). The population consisted of registered members of the Norwegian Nurses Organization (NNO) who held at least a 50% full time equivalent working position. At baseline 50.3% of the nurses reported holding a >90% full time equivalent position. NNO includes most of the working nurses in Norway. Written consent were obtained from all participants. At baseline assessment in 2008/2009, 5,400 nurses received a questionnaire, and 2,059 responded, yielding a response rate of 38.1%. After the first initial round was conducted, an additional group of newly educated nurses was invited to the cohort in order to increase the study population. Consequently, 2,741 new nurses received the baseline questionnaire, whereof 906 responded (response rate 33.1%). Thus, the total number of respondents in the first wave consisted of 2,965 nurses. These made up the cohort who were asked again at follow-up unless they for some reason had quit the study (*n* = 162). At follow-up after 6 years 1,892 nurses responded, yielding a response rate of 67.5% (1,892/2,803). In the present study, we excluded nurses who were pregnant at baseline or at follow-up and included only those nurses who reported their work schedule in both questionnaires. This final sub-cohort consisted of 1,371 nurses.

### Data

The following data were extracted for the present study:

From baseline: Sex, age, whether the participants had children living at home, and years since graduation. The following were extracted at both time points: Work schedule, self-reported number of quick returns worked the previous year, self-reported number of night shifts worked the previous year, exercise habits, caffeine consumption, smoking habits, alcohol habits, and pregnancy status. Total follow-up time was 6 years.

### Work Schedule

Participants were asked about their work schedule: Day only, evening only, two-shift rotation (day and evening), three-shift rotation (day, evening and night), night only, or another schedule including night work.

**Figure 1:**
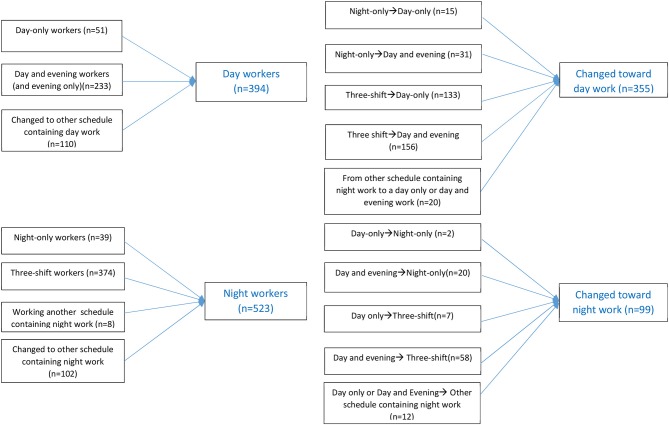
An overview of the four work schedule groups.

Those who had the same schedule at both baseline and follow-up were first regarded as separate groups: Day only (*n* = 51), day and evening (*n* = 233), night only (*n* = 39), and three-shift work (*n* = 374). Due to the small group sizes, the day only workers, day and evening workers, and those nurses who changed schedule but maintained a schedule without night work (*n* = 110) in the follow-up period were collapsed into a single group of day workers (*n* = 394). Similarly, night only workers (*n* = 39), three shift workers (*n* = 374), and those who worked another schedule containing night work (*n* = 8) and those who changed but maintained a scheduled containing night work (*n* = 102) were collapsed into a group of night workers (*n* = 523). We classified those who changed from a schedule containing night work into a schedule with day and/or evening work into one subgroup (*n* = 355). Furthermore, we classified those who changed toward a schedule containing night work from a schedule containing only day and/or evening shifts into another subgroup (*n* = 99). Thus, for our main analysis we had a total of four groups (*n* = 1,371): day workers, night workers, and those who changed toward- or away from a schedule containing night work. Figure 1 shows an overview of the four work schedule groups.

Typical work hours for nurses in rotational work schedules in Norway are 07:00–15:00 (day shift), 14:30–22:00 (evening shift), and 22:00–07:00 (night shift). There may be local variations, especially among day only workers in outpatient clinics, where for example 08:00–16:00 shifts are quite frequent. Shift workers in full position in Norway most often have a 35.5 h work-week, whereas day only workers in full position have a 37.5 h-work week.

### Average Number of Yearly Quick Returns (QR)

At both baseline and follow-up the nurses were asked about their number of QR the last year. We used these numbers to calculate an average from the two timepoints. This average was used in the statistical analyses as a proxy for average number of yearly QR in the follow-up period. The continuous variable was categorized into three subgroups where the lowest group was chosen as contrast. We minimized the exposure in the reference group while at the same time keeping a sufficient group size: <5 QR (*n* = 172), 5–35 QR (*n* = 535), >35 QR (*n* = 583). In order to investigate the effect of the magnitude of change in QR exposure between baseline and follow-up we made a change score using those shift workers with the lowest change scores as contrast: ±10 difference in number of QR between baseline and follow-up (*n* = 435), >10 decrease in number of QR (*n* = 454), and >10 increase in number of QR (*n* = 401).

### Average Number of Yearly Night Shifts (NN)

At both baseline and follow-up, the nurses were asked about their number of night shifts the last year (NN). As for QR we used this to calculate an average from the two timepoints. This average was used in the statistical analyses as a proxy for the average number of yearly night shifts in the follow-up period. We categorized this continuous variable into three subgroups where the lowest group was chosen as contrast. Again, we tried to minimize exposure in the contrast group while also keeping a sufficient group size: <1 NN (*n* = 289), 1–20 NN (*n* = 568), and >20 NN (*n* = 493). As for QR, we also investigated change from baseline to follow-up for NN: ±10 difference in number of NN between baseline and follow-up (*n* = 668), >10 decrease in NN (*n* = 392), and >10 increase in NN (*n* = 290).

### Exercise

At both baseline and follow-up the nurses were asked about exercise as measured by an item about hours of sweaty exercise per week (0, <1 h, 1–2 h, ≥3 h). This item was dichotomized (<1 h and ≥1 h per week). Additionally, those who exercised the least (0 h) were compared to those who exercised the most (≥3 h) using a separate dichotomized variable. The question concerning exercise used in the present study has been compared to V02_max_ and activity sensor and was found to be a reasonably valid measure of vigorous activity (27). Regarding the cut-off, one study reported that at least 1 h walking per week predicted lower cardiovascular risk. And, in addition, that vigorous activity predicted lowest risk (comparing highest to lowest categories) (28).

### Caffeine

At both baseline and follow-up the nurses were asked to estimate average number of caffeine containing units consumed per day and to report this as a continuous variable. Caffeine consumption was evaluated as a dichotomous parameter (drinking three or more caffeine containing units vs. <3 units per day). An umbrella review of meta-analyses suggested that the optimal risk reduction for various health outcomes was found for intake of 3–4 cups of coffee per day (29). Another large epidemiological study found that lower mortality was observed for all groups of those consuming coffee compared to non-drinkers (30). A significant trend was found for both male and female coffee drinkers: those consuming 2–3 cups of coffee per day or more had reduced mortality than those with lower consumption (30).

### Smoking

Smoking prevalence was assessed at both baseline and follow-up. The nurses were asked “do you smoke daily now (yes/no)?” Furthermore, those who smoked were in addition asked “If you smoke daily, how many cigarettes do you smoke per day?”

### Alcohol

At both baseline and follow-up, alcohol consumption and habits were evaluated using the short form of the Alcohol Use Disorders Identification Test Consumption (AUDIT-C). AUDIT-C is a self-report instrument with three items. The instrument appears to be a practical, valid primary screening test for heavy drinking, and/or active alcohol abuse or dependence (31). A score of 3 or higher on the AUDIT-C might indicate potential alcohol misuse. In a primary care setting a threshold score of 3 in females, and 4 in males maximized sensitivity and specificity (32). In our analysis, we used the AUDIT-C score both as a continuous as well as a dichotomous parameter (AUDIT-C cut off: ≥3 for females and ≥4 for males). For AUDIT-C we only had complete and accurate baseline and follow-up measurements of the sub-cohort of newly educated nurses.

### Statistical Analysis

SPSS, version 25 was used for the analyses. Continuous variables were expressed as means (±SD) or median (IQR) and categorical variables as proportions (%). For demographic data among different work schedules, ANOVA and Kruskal-Wallis Test were used to compare means/medians, and chi-square tests were used to compare proportions. The lifestyle factors were analyzed for both within and between group changes in the follow-up period when investigating day workers, night workers, those who changed away from a schedule containing night work, and those who changed toward a schedule containing night work. For evaluating within-group changes in different work schedules we compared continuous variables using paired *t*-tests, and proportions by McNemar's tests.

The relationship between the individual dependent variables (exercise, caffeine, smoking, alcohol) and the collapsed work schedule groups were studied using logistic regression. In addition, the original groups, the day only workers, day and evening workers, night only workers, and three-shift workers were evaluated in the logistic regression models. In addition to adjusting for age and sex, we adjusted for years since graduation because of possible work-related effects (e.g., experience) beyond our follow-up period, children living at home (yes/no) due to the potential for non-work related disruption of work-life balance and sleep, and baseline values of the respective dependent variable. Work schedule was dummy coded using day workers as a contrast, and the other work schedules were compared separately to the day workers. For average number of yearly QR and NN we used the groups with the lowest exposure as contrasts when evaluating the individual dependent variables in the same manner as for the four defined work schedules. The same was done for the change score variables for QR and NN. Significance level was set to *p* < 0.05.

### Ethics

The project was approved by the Regional Committee for Medical and Health Research of Western Norway (REK-WEST) (NO. 088.88).

## Results

### Demographics

In this study of 1,371 nurses, the mean age was 32.6 years [standard deviation (SD) = 8.5 years] at baseline. The study population consisted predominately of females (89.6%). At baseline, mean years since graduation were 3.8 years (SD = 4.1). 68.5% (*n* = 935) reported being in a relationship and 45.6% (*n* = 602) reported having children living at home. At baseline, three-shift rotation was most common (52.6%, *n* = 721), followed by two-shift rotation (30.6%, *n* = 420), night only (8.4%, *n* = 115), day only (5.3%, *n* = 72), and other schedules including night work (3.1%, *n* = 42), respectively. Only one person reported working evening only. At baseline, 69.6% of the nurses reported working in a somatic hospital department, 13.1% in a psychiatric service, 8.0% in nursing homes, 6.6% in home care services, and 2.7% in other positions. Mean number of average yearly quick returns was 31.3 (range 0–171, SD = 23.6). Mean number of average yearly night shifts was 21.2 (range 0–215, SD = 25.8). An overview of the distribution on these variables for day workers, night workers, and those who changed work schedule toward- or away from a work schedule containing night work is provided in Table 1.

**Table 1:** Demographics of Norwegian nurses with different work schedules in the 6-year follow-up period (*n* = 1,371).

	**Day workers (*****n*** **=** **394)**			**Night workers (*****n*** **=** **523)**			**Stopped worki*****n*****g nights (*****n*** **=** **355)**			**Started working nights (*****n*** **=** **99)**			***P*-value**
Sex (% female)^a^	*N* = 392	90%		*N* = 521	88%		*N* = 355	91%		*N* = 98	91%		0.56^b^
Age^a^ mean (SD)	*N* = 392		34.7 (9.3)	*N* = 522		31.5 (7.6)	*N* = 355		32.5 (8.4)	*N* = 99		30.4 (8.0)	**<0.001^c^**
Children living at home (% yes)^a^	*N* = 379	51%		*N* = 506	43%		*N* = 341	47%		*N* = 93	36%		**0.02**^**b**^
Years since graduation^a^ media*n* (IQR)	*N* = 393		2.0 (0.0-8.0)	*N* = 521		3.0 (0.0-7.0)	*N* = 354		3.0 (0.0-7.0)	*N* = 99		1.0 (0.0-3.0)	**<0.001^d^**

a *Data recorded at baseline*.

b *Evaluated using Pearson Chi-Square*.

c *Evaluated using one-way ANOVA*.

d *Evaluated using Kruskal-Wallis Test*.

*N, number of individuals included in the analysis; SD, Standard Deviation; IQR, 25–75 percentiles*.

*Bold values represent p < 0.05*.

### Exercise

We did not find any significant differences when analyzing the four defined work schedule groups and exercise (<1 h and ≥1 h per week) for within-group changes in the follow-up period (Table 2). Regarding the three predictors (work schedule, QR and NN), we did not find any significant differences among the subgroups concerning exercise habits (Table 3). Neither did we find any differences when comparing those workers who exercised the least to those who exercised the most (data not shown). Furthermore, no differences were found between the change score variables for QR and NN and exercise (data not shown).

**Table 2:** Baseline and 6 year follow-up values of lifestyle factors among Norwegian nurses with different work schedules (*n* = 1,371).

	**Day workers (*****n*** **=** **394)**				**Night workers (*****n*** **=** **523)**				**Stopped working nights (*****n*** **=** **355)**				**Started working nights (*****n*** **=** **99)**			
	***N***	**Baseline (SD)**	**Follow-up (SD)**	***P*-value**	***N***	**Baseline (SD)**	**Follow-up (SD)**	***P*-value**	***N***	**Baseline (SD)**	**Follow-up (SD)**	***P*-value**	***N***	**Baseline (SD)**	**Follow-up (SD)**	***P*-value**
Exercise habits (≥1 h/week)^a^	364	65%	63%	0.40	493	67%	64%	0.42	331	65%	62%	0.28	95	74%	71%	0.70
Caffeine consumptio*n* (units/day)^b^	391	3.2 (2.5)	3.8 (2.4)	**<0.001**	520	3.1 (2.5)	3.7 (2.8)	**<0.001**	352	2.8 (2.2)	3.5 (2.2)	**<0.001**	99	2.6 (2.2)	3.4 (2.2)	**<0.001**
≥3 units/day^a^	391	54%	69%	**<0.001**	520	55%	69%	**<0.001**	352	51%	69%	**<0.001**	99	48%	64%	**<0.001**
Smoking prevalence (%yes)^a^	378	17%	11%	**0.003**	508	11%	7%	**0.003**	239	7%	5%	0.31	97	11%	5%	0.07
Number of cigarettes/day^b^^,^^c^	63	9.1 (5.1)	3.9 (5.5)	**<0.001**	57	8.7 (5.3)	4.3 (6.4)	**<0.001**	23	9.0 (4.1)	3.1 (4.9)	**<0.001**	11	10.9 (5.9)	3.6 (5.5)	0.09
Average AUDIT-C score^b^	121	2.8 (1.9)	3.2 (1.8)	**0.04**	135	3.3 (0.19)	3.2 (1.8)	0.96	93	3.4 (1.7)	3.1 (1.7)	0.20	43	3.2 (1.8)	3.4 (1.9)	0.33
Above screening threshold^a^	121	58%	62%	0.51	135	64%	64%	1.00	93	73%	65%	0.19	43	61%	65%	0.79

a *McNemar's test for paired proportions. Proportions as percentages (%)*.

b *Paired t-test for continuous variables. Means (SD, Standard deviation)*.

c *Among smokers*.

*N, number of individuals included in the analysis*.

*Bold values represent p < 0.05*.

**Table 3:** Logistic regression models evaluating lifestyle factors among Norwegian nurses (*n* = 1,371) with respect to work schedules, average number of yearly quick returns and average number of yearly night shifts at 6-year follow-up.

	**Exercise habits (≥1 h/week)**		**Caffeine consumption (≥3 units/day)**		**Smoking prevalence**		**Alcohol consumption (above screening threshold)**	
	**Crude** **(*N* = 1283/1205/1263)** **OR (CI)**	**Adjusted** **(*N* = 1224/1152/1205)** **OR (CI)**	**Crude** **(*N* = 1362/1281/1341)** **OR (CI)**	**Adjusted** **(*N* = 1299/1224/1279)** **OR (CI)**	**Crude** **(*N* = 1309/1231/1290)** **OR (CI)**	**Adjusted** **(*N* = 1250/1178/1231)** **OR (CI)**	**Crude** **(*N* = 392/374/388)** **OR (CI)**	**Adjusted** **(*N* = 375/359/371)** **OR (CI)**
**WORK SCHEDULE**								
Day workers (contrast)	1.0	1.0	1.0	1.0	1.0	1.0	1.0	1.0
Night workers	1.06 (0.79–1.43)	1.11 (0.82–1.52)	0.96 (0.69–1.33)	1.01 (0.71–1.42)	0.75 (0.44–1.28)	0.86 (0.50–1.51)	0.96 (0.55–1.67)	1.32 (0.73–2.41)
Stopped working *n*ights	0.96 (0.69–1.32)	0.99 (0.71–1.38)	1.10 (0.76–1.57)	1.10 (0.76–1.60)	0.68 (0.36–1.31)	0.76 (0.39–1.50)	0.84 (0.46–1.55)	1.02 (0.54–1.94)
Started working nights	1.30 (0.78–2.17)	1.47 (0.85–2.54)	0.86 (0.51–1.47)	1.07 (0.61–1.87)	0.50 (0.17–1.41)	0.61 (0.21–1.79)	1.16 (0.51–2.45)	1.43 (0.63–3.26)
**AVERAGE NUMBER OF YEARLY QUICK RETURNS**								
<5 (contrast)	1.0	1.0	1.0	1.0	1.0	1.0	1.0	1.0
5–30	1.00 (0.68–1.48)	1.10 (0.74–1.65)	0.95 (0.61–1.46)	0.98 (0.62–1.53)	1.03 (0.46–2.27)	1.26 (0.54–2.95)	0.70 (0.31–1.58)	0.73 (0.32–1.67)
>30	0.95 (0.64–1.39)	1.02 (0.68–1.53)	1.12 (0.72–1.73)	1.15 (0.73–1.81)	1.05 (0.48–2.29)	1.24 (0.54–2.86)	0.95 (0.42–2.15)	1.02 (0.44–2.36)
**AVERAGE NUMBER OF YEARLY NIGHT SHIFTS**								
<1 (contrast)	1.0	1.0	1.0	1.0	1.0	1.0	1.0	1.0
1–20	0.84 (0.61–1.16)	0.90 (0.64–1.26)	1.25 (0.87–1.77)	1.35 (0.93–1.96)	0.88 (0.50–1.55)	0.95 (0.52–1.71)	0.95 (0.52–1.7)	1.19 (0.63–2.25)
>20	1.04 (0.74–1.45)	1.06 (0.74–1.50)	1.20 (0.83–1.73)	1.33 (0.90–1.95)	0.62 (0.34–1.13)	0.73 (0.39–1.37)	0.93 (0.48–1.78)	1.28 (0.63–2.60)

*In the crude model only baseline values of the respective dependent variables were adjusted for. Age, sex, years since graduation, children living at home or not, and baseline values of the respective lifestyle factors were covariates in the adjusted model. In addition, the original work schedules were analyzed using day only workers as contrast: no significant differences were found. N, number of individuals included in the analysis (work schedule/average number of yearly quick returns/average number of yearly night shifts); OR = Odds Ratio, CI = 95% Confidence Interval*.

### Caffeine

For all four work schedule groups there was an increase in caffeine consumption. The increase in caffeine consumption from baseline to follow-up was significant both when caffeine consumption was measured as a continuous parameter (units/day) and as a dichotomous parameter (≥3 units/day) (Table 2). We did not find any significant differences in caffeine consumption in our crude or adjusted logistic regression models between different work schedules, QR or NN groups (Table 3). Furthermore, no differences were found between the change score variables for QR and NN in terms of caffeine (data not shown).

### Smoking

Smoking prevalence decreased significantly in the follow-up period for both day and night workers (Table 2). For the two work schedule groups that stopped or started working nights there was a non-significant decrease in smoking prevalence. For all groups, except those who changed to a work schedule including night work, there was a significant decrease in number of cigarettes smoked per day among the smokers in the follow-up period (Table 2). We did not find any between-group differences in our logistic regression models with respect to smoking prevalence for different work schedules, OR or NN (Table 3). Furthermore, no differences were found between the change score variables for QR and NN and smoking (data not shown).

### Alcohol

Day workers were the only group with a significant increase in their AUDIT-C score (Table 2) in the follow-up period. We did not find any significant between-group changes with respect to alcohol consumption for different work schedules, QR and NN (Table 3). Furthermore, no differences were found between the change score variables for QR and NN and AUDIT-C (data not shown).

### Additional Analyses

We also analyzed the original work schedule groups (day only (contrast); day and evening; night only; three-shift rotation) in separate logistic regression models for each of the lifestyle factors. No significant differences were detected (data not shown).

## Discussion

To the best of our knowledge this is one of few papers that addresses the relationship between shift work and lifestyle factors using a prospective design. This paper investigated different work schedules, different exposures to QR and different exposures to NN during a 6-year follow-up. We found a significant increase in caffeine consumption in all four defined work schedules. However, we did not find any differences in lifestyle factor trajectories across the different work schedules or across differences in exposure to QR or NN.

A significant increase in caffeine consumption across all work schedules was found in the present study. Several studies have found positive effects of caffeine concerning increased performance and alertness and that caffeine could be an effective intervention to mitigate sleepiness and prevent injuries and errors (33–35). The increase found in our study might be due to nurses using caffeine to enhance alertness and mitigate sleepiness or as a result of a general increased consumption with age (36). However, we did not find any longitudinal relationship between caffeine consumption and different work schedules, QR or NN. However, the findings are consistent with Drake et al. who did not find any significant difference between day workers, permanent night workers, or rotating shift workers concerning caffeine intake in a cross-sectional study (37). In contrast, Ramin et al. found a significantly higher caffeine intake when comparing those who had always worked nights with those who had never worked night shifts (38). Both early morning shifts and night shifts may be challenging for nurses since both shift-types may interfere with the nurses' individual circadian rhythms and thus result in high levels of sleepiness. The same argument could be valid for QR and NN. High exposure of QR or NN could potentially leave the shift worker in constant circadian misalignment, challenged by conflicting work and domestic demands. Still, caffeine consumption was not higher in these subgroups of nurses.

Concerning exercise, we did not find any clear differences between the four defined work schedules, different exposure to QR or different exposure to NN in the follow-up period. Our measurement of heavy exercise might be too crude to detect any minor difference between groups. However, several former studies have looked at shift work and exercise, and overall no clear differences have been found (16, 39, 40). Loef et al. reported that shift workers spend more time walking but found no difference among shift workers and non-shift workers with regards to other non-occupational physical activities (39). Other studies have also found shift workers not to differ from day workers in terms of leisure time physical activity, but shift workers seem to have a lower activity level at work (16, 40). While not finding any differences in physical activity between day and shift workers, Kiwimaki et al. still found higher rates of obesity among shift workers than day workers (16). This is consistent with previous studies from this same cohort among Norwegian nurses (41, 42). Since the present and previous studies do not report any significant differences in physical activity levels between day and shift workers, one may speculate that the observed differences in weight and weight gain might be due to differences in the distribution and the temporal changes in eating habits or changes in metabolism due to circadian disruption and insufficient sleep (11, 43).

The overall decline in smoking prevalence was probably not unique to our cohort and probably reflects preventive measures and increased health awareness in the general population. According to Statistics Norway, the smoking prevalence of females in Norway decreased from 22 to 10% between 2007 and 2017 (44). Ramin et al. found a higher smoking prevalence among ever night workers compared to never night workers (38). However, the study did not have a prospective design and could thus not evaluate trends in smoking prevalence between the different groups. A few studies have taken a different approach and looked at smoking cessation and the proportion of workers starting smoking. Van Amelsvoort et al. found higher odds of being a smoker among shift workers compared to day workers at baseline. Furthermore, the follow-up also revealed that shift workers were more prone to start smoking compared with day workers (45). This finding is consistent with a Danish study which found fixed night workers to have a higher odds of smoking relapse and lower odds of smoking cessation compared to fixed day workers (46). However, we found no significant differences in smoking between the work schedules or in relation to exposure to QR and NN.

Day workers were the only group with a significant within-group increase in the AUDIT-C score at follow-up. Thus, our hypothesis that shift work would affect this habit adversely was not supported. Using AUDIT-C as a dichotomous parameter (under/above screening threshold), we neither found any significant longitudinal difference across different work schedules nor concerning QR and NN exposure. This is consistent with another study with respect to total alcohol intake (16). Similarly, Morikawa et al. did not find any differences between day workers or shift workers in the volume of alcohol consumption or heavy drinking. However, Morikawa et al. found the highest frequency of heavy drinking in a subgroup of night workers sleeping poorly, leading the authors to suggest that alcohol might be used as a sleep aid (47).

The strengths of this study were its large sample size, the prospective design, and evaluation of different aspects of shift work that might contribute to altered health behaviors. Also, the relatively wide range of lifestyle factors (exercise, caffeine, alcohol, and smoking) constitutes a strength. We believe that potential long-term changes in lifestyle factor trajectories could be of clinical importance, for example, concerning cardiometabolic health. Our follow-up period over 6-years is thus one of the major assets of the present study. It should also be noted that we have addressed some of the limitations in other studies as reviewed by Proper et al. by employing a large prospective design, evaluation of different aspects of shift work, and investigation of the possible mediating role of lifestyle factors (21). The present cohort was relatively young and relatively newly educated. One could argue that lifestyle factor trajectories over time might not have a linear but a curve-linear relationship, consequently, changes in lifestyle factors could attenuate over the relatively long follow-up time. From such a viewpoint it might be a strength that this cohort comprises relatively newly educated nurses.

Our study relies on self-reported data that may have uncertainties and potential for different kind of biases. Concerning recall bias, the data used in the present study were collected with a maximum of 1-year recall. Brisson et al. found that self-reported data collected close to specific events are highly accurate and have high validity (48). Due to small group sizes among day only and night only workers, the original work schedule groups were collapsed into one group for nurses without night work and one group for nurses with night work in the follow-up period. This was done to ensure sufficient group size and statistical power, and at the same time still be able to compare nurses with night work to those without night work. Still, this is a limitation and caution is warranted in interpreting the results. However, we did also analyze the original working schedules (day only, day and evening, night only, three-shift rotation) without finding any significant differences. Obviously, a limitation of this approach was the small group sizes of some of the working schedules. Still, similar findings shown when collapsing work schedule groups strenghten our conclusion. We investigated those who changed toward- or away from night work during the follow-up period. A limitation here is that we did not account for when they changed schedule. Regarding QR and NN as exposure variables, most of the nurses worked regular schedules and should thus be able to make good estimations of the magnitude of these variables on a yearly basis. When comparing different levels of exposure, we wanted the contrast group to have low exposure to night shifts and quick returns. It was however not possible to have contrast groups with no exposure, since very few nurses reported no QR or no NN. However, we will argue that the exposure in the contrast groups (<5 QR/year and <1 NN/year) was still very low and makes as such an adequate contrast. Due to how the schedules are organized and that many nurses work extra shifts almost all nurses were exposed to QR. We therefore had to have a different cut-off in the contrast group for QR compared to for NN. We cannot rule out the possibility of an uneven exposure to these two parameters in the follow-up period. Nurses moving from high to low, or low to high exposure of these two parameters may be an important group due to selection effects. We tried to address this by doing separate analyses comparing those with a stable exposure to those who increased or decreased their exposure to QR and NN, respectively. Still, the results remained the same.

One may question the generalizability of our study, as the cohort was based upon Norwegian nurses, most of them being female. The results are still likely to be valid for all Norwegian nurses, as the study was based upon a sample of the total population in this country. However, the results might be different in other occupational groups. Also, the results may not be valid for other countries, as working conditions and e.g., smoking regulations are different from country to country. In terms of smoking decline, there has since 1998 been a legal protection from exposure to smoking in workplaces in Norway, only allowing smoking in separate smoking rooms (49). Norway, like all Scandinavian countries, is a welfare state and has well-organized and regulated work environments with relatively few working hours in a full-time equivalent work week (35.5–37.5 h), which may limit generalizability. Another limitation concerning the measure of alcohol habits in the present study was that we only had complete and accurate data for a subgroup of the nurses. If this subgroup is not representative of the whole cohort population, this may thus limit generalizability. The AUDIT-C is a validated screening tool with 3 questions about potential alcohol misuse. A limitation is that while the two first questions address frequency and volume, we do not have exact information about daily or weekly alcohol consumption, for example units/week. The data may, thus, fail to detect nuances and changes in lighter or normal alcohol consumption habits which could be of importance. One of the inclusion criteria in the SUSSH cohort was that nurses had to hold at least a 50% full time equivalent working position. Still, there will be variations in their weekly hours. This could be a limitation, especially for working schedules which do not account for this. However, it should also be noted that many nurses with smaller permanent positions work extra shifts which are not accounted for in the data. Concerning NN and QR exposure, these parameters are reported as a continuous parameter and should thus reflect the nurses' actual exposure.

In this cohort, many of the nurses changed their work schedule away from night work (*n* = 355). Another study found that between 8 and 35%, depending on their type of shift work, changed to day work during a 6-year follow-up (50). The selection biases within shift work could be seen as a “healthy worker effect”: It is more likely that healthy workers tend to choose and stay in a challenging work schedule (22). This could potentially underestimate the real effect of shift work on lifestyle factors trajectories. Another potential for underestimation of the true effects is misclassification bias. Härmä et al. when comparing self-reported data to objective registry data, found that for those who reported working shift work without night shifts there was a low sensitivity (62%) due to the fact that many nurses worked nights but did not report this (50). The authors concluded that this exposure misclassification was likely to bias results. Misclassification bias could be present in our study and could be a source of underestimation of true effects.

Our study had a low initial response rate, but a high response rate at follow-up. A review by Baruch et al. suggested that most study populations have a response rate about 53 ± 20% (1 SD from the mean response rate) (51). The low response rate in the first wave might have resulted in a skewed sample, but this is of less importance in the present study where we looked at changes over time.

## Conclusion

We did not find any differences in relation to different work schedules, different exposure to QR, or different exposure to NN concerning exercise, caffeine consumption, smoking prevalence, and alcohol consumption in this 6-year follow-up study. This suggests that shift work may not affect lifestyle factors adversely and challenges the notion that shift work has an adverse impact on lifestyle factors. More prospective studies are needed to verify our findings.

## Data Availability Statement

The anonymized data supporting the conclusions of this manuscript will be made available by the authors, without undue reservation, to any qualified researcher.

## Author Contributions

HB: design of the study, data analysis, interpretation of the results, drafting the paper. SP, SW, BM, and BB: collecting the data, design of the study, interpretation of the results, critical review of the paper. All authors have approved the final manuscript.

### Conflict of Interest

The authors declare that the research was conducted in the absence of any commercial or financial relationships that could be construed as a potential conflict of interest.
